# Supplementary figures and images for: Identification of miRNA Master Regulators in Breast Cancer
Source: Cells. 2020 Jul 3;9(7):1610. doi: 10.3390/cells9071610 (PMC7407970; doi:10.3390/cells9071610)

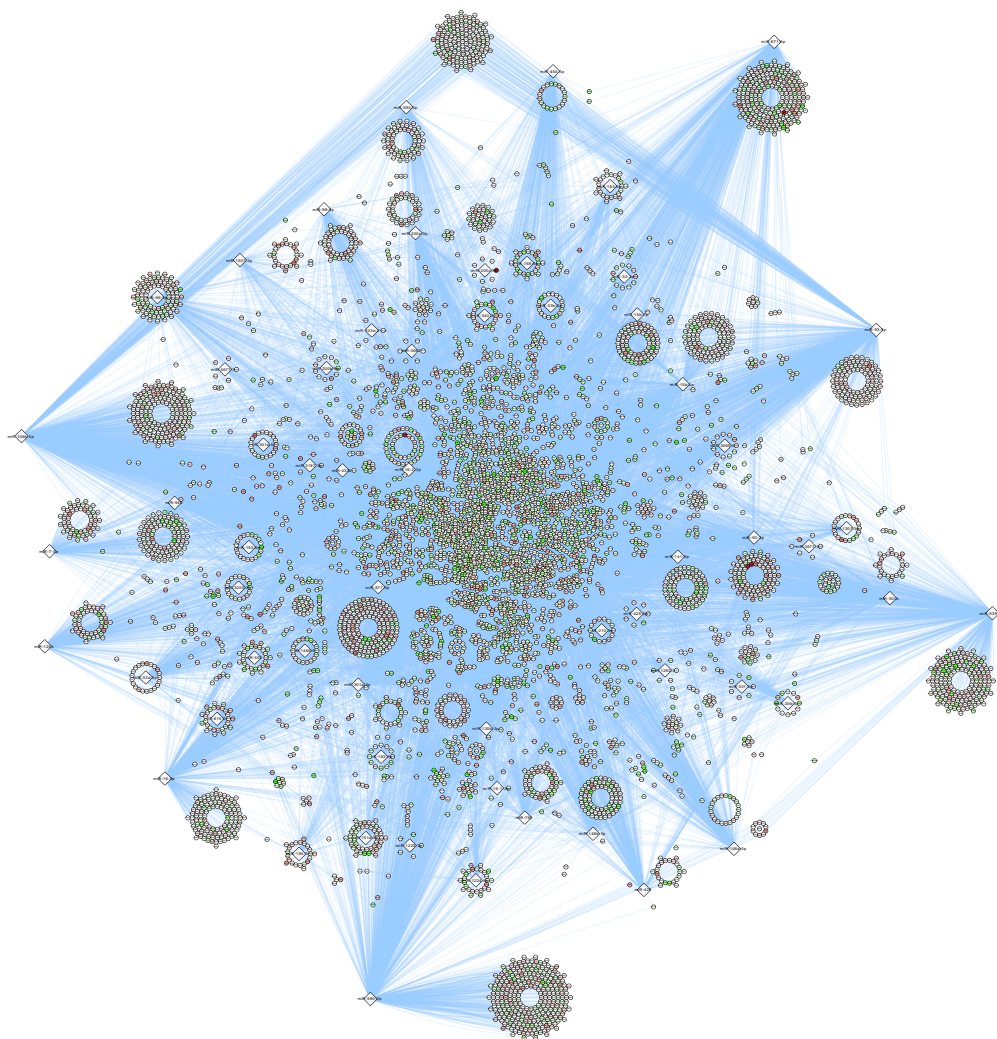

Supplement: Supplementary file 1 [file cells-09-01610-s001.zip › SUPLEMENTARIAS/SUP_FIG1.pdf]
